# MASH in Type 2 Diabetes: Pathophysiology, Diagnosis, and Therapeutic Management—A Narrative Review

**DOI:** 10.3390/medicina62020325

**Published:** 2026-02-05

**Authors:** Adela Gabriela Ştefan, Adina Mitrea, Diana Clenciu, Ionela Mihaela Vladu, Maria Magdalena Roşu, Diana Cristina Protasiewicz-Timofticiuc, Theodora Claudia Radu-Gheonea, Ion-Cristian Efrem, Anca Maria Amzolini, Beatrice Elena Vladu, Ana-Maria Efrem, Delia-Viola Reurean Pintilei, Eugen Moţa, Maria Moţa

**Affiliations:** 1 Department of Diabetes, Nutrition and Metabolic Diseases, Calafat Municipal Hospital, 205200 Calafat, Romania; adela.firanescu@yahoo.com; 2 Department of Diabetes, Nutrition and Metabolic Diseases, Faculty of Medicine, University of Medicine and Pharmacy of Craiova, 200349 Craiova, Romania; adina.mitrea@umfcv.ro (A.M.); theodora.gheonea@umfcv.ro (T.C.R.-G.); 3 Department of Diabetes, Nutrition and Metabolic Diseases, Faculty of Midwives and Nursing, University of Medicine and Pharmacy of Craiova, 200349 Craiova, Romania; maria.rosu@umfcv.ro (M.M.R.); diana.protasiewicz@umfcv.ro (D.C.P.-T.); 4 Department of Internal Medicine—Medical Semiology, Faculty of Dentistry, University of Medicine and Pharmacy of Craiova, 200349 Craiova, Romania; cristian.efrem@umfcv.ro; 5 Department of Medical Semiology, Faculty of Medicine, University of Medicine and Pharmacy of Craiova, 200349 Craiova, Romania; anca.amzolini@umfcv.ro; 6 Faculty of Medicine, University of Medicine and Pharmacy of Craiova, 200349 Craiova, Romania; beatricevladu75@gmail.com; 7 Faculty of Medicine, University of Medicine and Pharmacy “Iuliu Haţieganu” Cluj-Napoca, 400012 Cluj-Napoca, Romania; anamaref23@gmail.com; 8 Department of Medical-Surgical and Complementary Sciences, Faculty of Medicine and Biological Sciences, “Stefan cel Mare” University, 720229 Suceava, Romania; delia.pintilei@usm.ro; 9 Consultmed Medical Centre, Department of Diabetes, Nutrition and Metabolic Diseases, 700544 Iasi, Romania; 10 Doctoral School, University of Medicine and Pharmacy of Craiova, 200349 Craiova, Romania; eugenmota@yahoo.com (E.M.); mmota53@yahoo.com (M.M.)

**Keywords:** metabolic dysfunction-associated steatohepatitis, metabolic dysfunction-associated steatotic liver disease, type 2 diabetes, metabolic syndrome, obesity

## Abstract

Metabolic dysfunction-associated steatotic liver disease (MASLD) has emerged as one of the greatest challenges for the modern public health system and serves as the foundation for the development of advanced stages, such as metabolic dysfunction-associated steatohepatitis (MASH), which may progress to fibrosis, cirrhosis, and hepatocellular carcinoma (HCC). MASLD and type 2 diabetes mellitus (T2DM) mutually exacerbate one another. MASLD increases the incidence of T2DM and the risk of complications in patients already affected. T2DM accelerates progression to MASH, which has become the second leading cause of liver transplantation and end-stage liver disease, and is associated with hepatic decompensation, cirrhosis, HCC, chronic kidney disease, and cardiovascular disease. MASLD and MASH are strongly linked to T2DM and obesity, pathogenesis including genetic polymorphisms, environmental factors, and multiple metabolic disturbances: insulin resistance (IR), gut dysbiosis, altered adipokine signaling, such as reduced adiponectin alongside increased pro-inflammatory cytokines. Inflammation plays a central role in the development of HCC in MASH, even in the absence of significant fibrosis. The Fibrosis-4 index (FIB-4) should be used as a first-line noninvasive tool to assess fibrosis risk. Additionally, ultrasound-based transient elastography (FibroScan) supports clinicians in assessing steatosis and fibrosis severity. Histologically, MASH is characterized by steatosis, lobular inflammatory changes, and ballooning degeneration of hepatocytes, with or without associated fibrosis. Accurately diagnosing and stratifying MASLD based on fibrosis risk is crucial to identify patients who may benefit from pharmacological treatment or can be managed only with lifestyle interventions. Patients should attain above 10% weight loss through lifestyle modifications. Resmetirom is recommended in F2/F3 fibrosis stages. For treating T2DM, glucagon-like peptide-1 receptor agonists and coagonists, sodium–glucose cotransporter-2 inhibitors, metformin (if glomerular filtration rate exceeds 30 mL/min), and insulin (in decompensated cirrhosis) are preferred. Clinical insights derived from trials are expected to optimize quality of life and long-term outcomes in patients with MASH.

## 1. Introduction

Hepatic steatosis was first described by Addison in 1836 [1,2,3], but it was not until 1980 that the term nonalcoholic steatohepatitis (NASH) was used. This term described a progressive form of hepatic steatosis, characterized by histological features resembling steatohepatitis associated with alcohol consumption, but occurring in individuals withoutexcessive alcohol consumption [1,4]. Subsequently, the term nonalcoholic fatty liver disease (NAFLD) was introduced six years later, in 1986 [1,5]. As interest in fatty liver pathology grew, the components of metabolic syndrome (MetS) were delineated, highlighting various metabolic disorders linked to NAFLD, including hypertriglyceridemia, insulin resistance (IR), and hyperinsulinemia [1,2,6]. Due to this strong interconnection, the nomenclature shifted again in 2020, when NAFLD was replaced by metabolic dysfunction-associated fatty liver disease (MAFLD), a change that introduced updated diagnostic criteria that allowed more permissive alcohol intake thresholds and removed the steatohepatitis subcategory [1,7].

However, some experts argued that the term “fat” carried stigmatizing connotations, which led to the introduction of the designation metabolic dysfunction-associated steatotic liver disease (MASLD) in 2023 [1,8]. With the growing prevalence of MetS, MASLD has emerged as one of the greatest challenges for the modern public health system, now representing the leading cause of chronic liver diseases [9,10,11,12]. MASLD spans a wide clinical spectrum and serves as the foundation for the development of more advanced stages, such as metabolic dysfunction-associated steatohepatitis (MASH), which may progress to fibrosis, cirrhosis, and hepatocellular carcinoma (HCC) [8,13].

MASLD currently affects an estimated 38% of the global adult population [1,14,15,16], along with a considerable proportion (7–14%) of adolescents and young adults [1,17]. Its prevalence is highest in Latin America (44.4%) and lowest in Western Europe (25.1%). Forecasts remain concerning, with MASLD global burden projected to reach 55.4% by 2040, largely fueled by the escalating incidence of type 2 diabetes mellitus (T2DM) and obesity [1,15,16,18,19]. Rapid increases in obesity and T2DM across North Africa, the Middle East, and Asia [1,20] have contributed to a MASLD prevalence of 39.43% in the general population and a truly alarming 68.71% among individuals with T2DM [1,21]. It is estimated that 20–30% of MASLD cases progress to MASH, with the prevalence of MASH in the general population projected to increase by 63% by 2030 [10,22]. MASH is likewise strongly linked to T2DM, with a prevalence of 37% in patients with T2DM, compared with 5–14% in the general population [10,14,23]. A recently published meta-analysis reported a MASLD prevalence of 65.33% among subjects with T2DM, with the highest rates observed in Eastern Europe (80.62%) and the Middle East (71.24%). The same study documented a MASH prevalence of 66.44% in this population, alongside substantial rates of significant fibrosis (40.78%) and advanced fibrosis (15.49%) [24].

MASLD and T2DM mutually exacerbate one another. MASLD not only increases the incidence of T2DM but also the risk of complications in patients already affected. Conversely, T2DM accelerates progression to MASH, advanced fibrosis, cirrhosis, and HCC [25,26,27]. MASH has become the second leading cause of liver transplantation and end-stage liver disease [25,28], and is associated with hepatic decompensation, cirrhosis, HCC, chronic kidney disease (CKD), and cardiovascular disease [11,29,30]. Consequently, the coexistence of MASLD and T2DM results in a markedly increased risk of adverse outcomes, with most deaths among patients with MASLD being attributed to cardiovascular events (40%), whereas only 4–8% are attributed to HCC and/or cirrhosis [14,31]. This review highlights recent advances in the epidemiology, pathogenesis, diagnosis approaches, and treatment of MASH in individuals with T2DM. This narrative review is based on a comprehensive evaluation of the available literature. Studies were identified through searches of PubMed and Scopus using combinations of keywords related to metabolic dysfunction-associated steatohepatitis (MASH), type 2 diabetes mellitus, and emerging therapeutic strategies. Priority was given to peer-reviewed clinical trials, meta-analyses, and high-quality observational studies published in English. Preclinical studies were included when relevant for mechanistic insight. Exclusion criteria included case reports, conference abstracts, editorials, non-peer-reviewed articles, and publications lacking sufficient methodological rigor or relevance to the scope of this review.

## 2. MASH Pathogenesis

**Figure 1:**
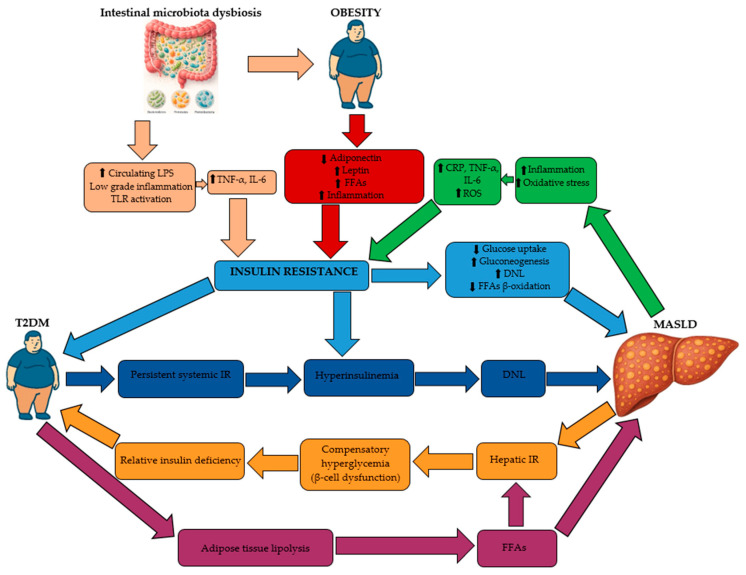
Pathophysiological mechanisms that link T2DM and MASLD. T2DM: type 2 diabetes mellitus; MASLD: metabolic dysfunction-associated steatotic liver disease; LPS: lipopolysaccharides; TLR: toll-like receptor; CRP: C-reactive protein; TNF-α: tumor necrosis factor alpha; IL-6: interleukin-6; ROS: reactive oxygen species; FFAs: free fatty acids; DNL: de novo lipogenesis; IR: insulin resistance; ↑: increased; ↓: decreased (adapted from [32]).

MASLD and MASH have reached pandemic proportions and are strongly linked to T2DM and obesity (Figure 1) [32].

**Figure 2:**
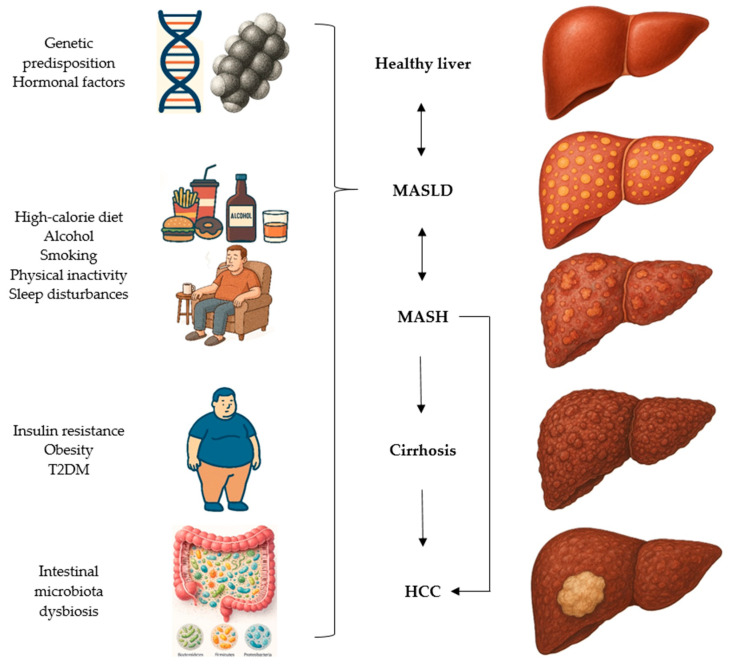
MASH pathogenesis and progression from a healthy liver to HCC. The initial phases of MASLD and MASH remain potentially reversible. MASLD: metabolic dysfunction-associated steatotic liver disease; MASH: metabolic dysfunction-associated steatohepatitis; HCC: hepatocellular carcinoma; T2DM: type 2 diabetes mellitus.

Current evidence favors the “multiple hits” model, indicating that the pathogenesis of MASH is far more intricate than the original hypothesis proposed by Day and James, which suggested that obesity leads to increased hepatic fat accumulation, requiring an additional “hit” to trigger inflammation and fibrosis [33,34,35]. The updated concept highlights a constellation of interacting factors, including genetic polymorphisms, environmental factors, and a spectrum of metabolic disturbances (Figure 2): IR, gut dysbiosis, and altered adipokine signaling. The latter is characterized by reduced adiponectin alongside increased pro-inflammatory cytokines, such as C-reactive protein (CRP), interleukin 6 (IL-6), and tumor necrosis factor alpha (TNF-α) [32,34]. These mechanisms enhance intestinal permeability, facilitating the translocation of bacterial endotoxins to the liver, thereby intensifying inflammation and promoting the onset of MASLD and its progression to MASH. In addition, the imbalance between reactive oxygen species (ROS) and antioxidant defenses induces oxidative stress, which further amplifies inflammation, fibrosis, and cellular death [33,36]. Inflammation plays a central role in the development of HCC in MASH, even in the absence of significant fibrosis, highlighting distinct pathogenic mechanisms compared with fibrosis-driven HCC [37,38,39].

### 2.1. Genetic and Epigenetic Risk Factors

In terms of genetic susceptibility, polymorphisms in the PNPLA3, HSD17B13, TM6SF2, MBOAT7, FATP5, and ApoC-III genes, alongside epigenetic changes, confer a higher risk for the onset of MASLD and its progression to MASH [25,33,40,41,42,43]. Epigenetic mechanisms modulate the expression of these genes, with chromatin remodeling and DNA methylation representing the key regulatory processes involved [33,44].

### 2.2. Hormonal Factors

The development of visceral adiposity is influenced by multiple mechanisms, including hormonal, physiological, and anatomical factors, with differences between sex-specific groups. Males typically display an android pattern, marked by increased visceral fat accumulation, whereas females exhibit a gynoid pattern, characterized by increased subcutaneous fat [45,46]. Estrogens play a crucial role in premenopause by reducing lipolysis that occurs in subcutaneous adipocytes [45,47], leading to substantial subcutaneous fat storage, along with enhanced lipoprotein lipase (LPL) activity in females [45,48]. Testosterone, in contrast, inhibits LPL activity [44,47]. Excess hepatic lipid accumulation primarily results from intensified visceral adipose tissue lipolysis, hepatic de novo lipogenesis (DNL) activation, and the consumption of hypercaloric or high-fat diets [49,50].

### 2.3. Diet and Lifestyle

High-calorie diets, especially those high in sugar and fat, lead to caloric surplus, promoting weight gain and hepatic fat accumulation [51]. In recent years, the Western diet, comprising ultra-processed foods such as fried items, pastries, sweets, and sugar-sweetened beverages, all rich in trans fats, saturated fats, and overall caloric density, has been linked to enhanced hepatic fat accumulation and stimulation of hepatic lipogenesis. These effects have been observed especially in diets high in fructose [33,52]. Such dietary patterns contribute to MASLD and MASH progression by inducing metabolic alterations, including IR, inflammation, increased visceral adiposity, and obesity [53]. Reduced physical activity further exacerbates IR and obesity [33]. Additionally, moderate alcohol consumption, smoking, and sleep disturbances markedly elevate the risk of developing MASH [54,55].

### 2.4. Insulin Resistance

IR is a central contributor to MASLD development [45,56], inducing adipose tissue’s resistance to insulin’s antilipolytic effects, resulting in elevated circulating free fatty acids (FFAs), which are subsequently stored in the liver as triglycerides (TG) [49,57]. To mitigate FFAs-induced toxicity, adipose tissue responds through hypertrophy and hyperplasia, activating common inflammatory pathways and macrophages and generating lipotoxic lipids. This cascade triggers cellular stress, promotes inflammation, leads to tissue remodeling, and ultimately to fibrogenesis [22,58,59]. Lipotoxicity, exacerbated by the effects of IR, correlates with both steatohepatitis severity and hepatic fibrosis, explaining the high prevalence of hepatosteatosis and moderate-to-advanced fibrosis in individuals with T2DM [22,45,60].

### 2.5. Intestinal Microbiota

Several factors, including metabolic disorders, diet, and comorbid conditions, can alter the composition of the intestinal microbiota, which in turn modulates energy balance by altering the absorption of calories from food and alcohol [42,61]. Individuals who develop MASH exhibit a reduced amount of beneficial bacteria, such as Bacteroidetes and Firmicutes, alongside an overrepresentation of Proteobacteria and Enterobacteriaceae [62]. In patients with MASH and MASLD, the gut microbiota often generates elevated levels of endotoxins, especially lipopolysaccharides, which enter the portal circulation and trigger hepatic inflammation [33,63]. Microbial metabolites, such as imidazole propionate and trimethylamine N-oxide (TMAO), promote metabolic dysregulation and exacerbate hepatosteatosis and fibrosis [33,64]. Additionally, bacterial components, such as flagellin and peptidoglycans, can activate the immune system, inducing the production of pro-inflammatory cytokines (IL-6, IL-1β, TNF-α), which stimulate hepatic inflammation and fibrosis [65,66].

## 3. MASH Diagnosis

**Figure 3:**
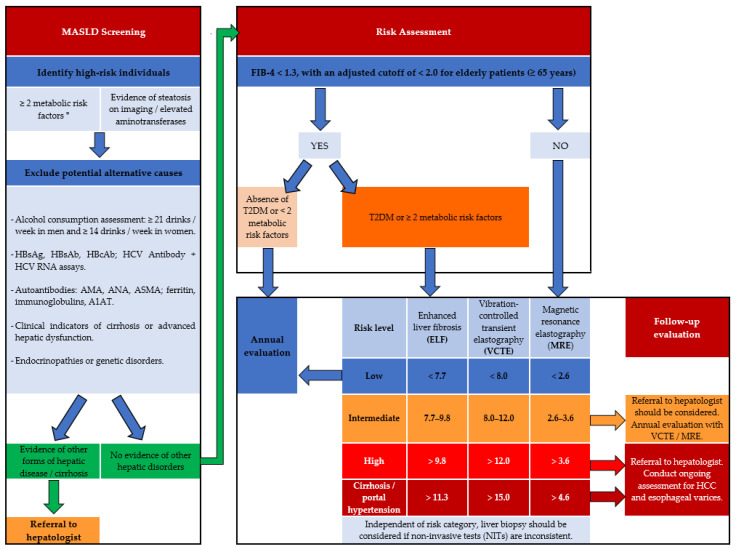
MASLD evaluation. MASLD: metabolic dysfunction-associated steatotic liver disease; T2DM: type 2 diabetes mellitus; HBsAg: hepatitis B surface antigen; HBsAb: hepatitis B surface antibody; HBcAb: hepatitis B core antibody; HCV: hepatitis C virus; RNA: ribonucleic acid; AMA: anti-mitochondrial antibody; ANA: antinuclear antibody; ASMA: anti-smooth muscle antibody; A1AT: alpha-1 antitrypsin; FIB-4: Fibrosis-4 index. * abdominal obesity, hyperglycemia, hypertension, hypertriglyceridemia, and decreased HDL-c (adapted from [67]).

MASLD frequently lacks clinical manifestations or manifests with nonspecific symptoms and is diagnosed based on evidence of ≥5% hepatic fat accumulation, in association with at least one of the following criteria (Table 1) [67,68,69]:

Routine yearly assessment for MASLD is recommended for individuals presenting with two or more metabolic risk factors, including obesity (particularly abdominal obesity), hyperglycemia, and dyslipidemia—defined by hypertriglyceridemia and decreased high-density lipoprotein cholesterol (HDL-c)— as well as hypertension and hypothyroidism [67,70,71]. To confirm MASLD, practitioners should first exclude potential liver disease etiologies. These include alcohol-related hepatotoxicity, viral hepatitis B or C— screened via hepatitis B surface antigen (HBsAg), hepatitis B surface antibody (HBsAb), hepatitis B core antibody (HBcAb), hepatitis C virus (HCV) antibody + HCV ribonucleic acid (RNA) assays—and autoantibodies, including anti-mitochondrial antibody (AMA), antinuclear antibody (ANA), anti-smooth muscle antibody (ASMA), and immunoglobulins. Furthermore, alpha-1 antitrypsin (A1AT), hemochromatosis, endocrinopathies (Cushing’s syndrome, hypothyroidism), or genetic disorders [67,71] should be ruled out. Patients with an elevated risk of fibrosis warrant referral to a hepatologist (Figure 3) [67,70,72].

The Fibrosis-4 index (FIB-4), derived from platelet count, alanine aminotransferase (ALT), aspartate aminotransferase (AST), and age, should be used as a first-line noninvasive tool to assess hepatic fibrosis risk associated with MASLD [67,72]. Individuals with FIB-4 values below 1.3 (or below 2.0 in those aged ≥65 years) who do not have T2DM or present fewer than two metabolic risk factors should undergo annual reassessment. In contrast, patients with elevated FIB-4 scores and/or T2DM or at least two metabolic risk factors require additional evaluation using the enhanced liver fibrosis (ELF) test and elastography, including vibration-controlled transient elastography (VCTE) or magnetic resonance elastography (MRE), to accurately stage hepatic fibrosis. Noninvasive diagnostic modalities are undergoing rapid development [67,73,74]. Patients with markedly abnormal results warrant referral to a hepatologist. Liver biopsy remains the gold standard for confirming MASH and is recommended when noninvasive procedures yield conflicting results [67].

An article published by our team demonstrated that elevated values of biomarkers such as TG, non-HDL-c-to-HDL-c ratio (NHHR), homeostasis model assessment of insulin resistance (HOMA-IR), and atherogenic index of plasma (AIP) are predictors for MASLD in patients with T2DM [75]. Furthermore, in two additional studies conducted by our team, we underscored the relevance of the triglyceride-glucose (TyG) index for the assessment of MASLD in patients with MetS, the biomarker levels showing a significant correlation with the severity of hepatic steatosis assessed by liver biopsy [76,77,78]. Nevertheless, it should be noted that while emerging biomarkers such as TyG, NHHR, and AIP show promising associations with MASH severity and cardiometabolic risk, the current evidence is largely derived from observational and hypothesis-generating studies. Further prospective validation in large, independent cohorts is required before their routine clinical implementation.

**Figure 4:**
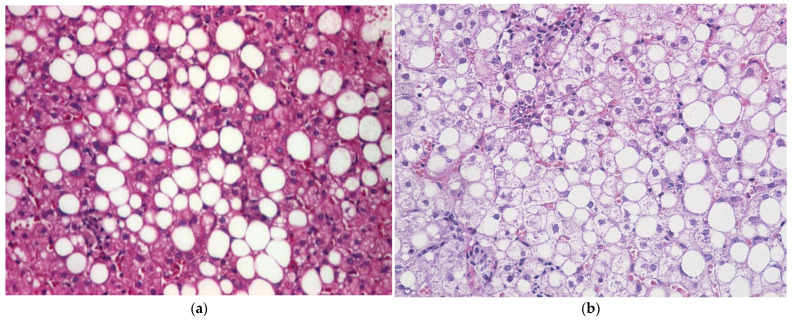
Liver biopsy images in patients with T2DM. (**a**) Macrovesicular steatosis characterized by large intracytoplasmic lipid vacuoles within hepatocytes, with peripheral displacement of nuclei. Hematoxylin–Eosin staining, ×200 (image courtesy of Ion Cristian Efrem). (**b**) Macrovesicular steatosis associated with mild intralobular inflammatory infiltrate and hepatocyte ballooning degeneration. Hematoxylin–Eosin staining, ×200 (image courtesy of Anca Maria Amzolini).

Histologically, MASH is characterized by hepatic steatosis, lobular inflammatory changes, and ballooning degeneration of hepatocytes, occurring with or without associated fibrosis (Figure 4) [79,80]. Patients with isolated steatosis experience a slower rate of fibrosis progression compared with those diagnosed with MASH, with an average progression of one fibrosis stage over 14.3 years versus 7.1 years, respectively [81,82]. However, approximately 20% of patients with MASH exhibit rapid progression to more severe forms of steatohepatitis and significant fibrosis [81]. Although fibrosis is not required for the diagnosis of MASH, its presence is the key prognostic factor for adverse outcomes in patients with MASLD, being strongly linked to increased liver-related mortality [31].

In light of the limitations, particularly those related to the risks and complications associated with liver biopsy, such as hemorrhage, procedural pain, hypotension, pneumothorax, visceral injury, and, in rare cases, mortality, the ultrasound-based transient elastography, commonly referred to as FibroScan, is an Food and Drug Administration (FDA)-approved technique that supports clinicians in assessing liver stiffness measurement (LSM), expressed in kilopascals (kPa), with values varying according to disease stage [45,83,84]. These values are closely associated with fibrosis severity. The assessment is based on an ultrasonic attenuation wave operating at 3.5 megahertz (MHz), while hepatic fat content is quantified using the controlled attenuation parameter (CAP), expressed in decibels per meter (dB/m) [45,85]. Accordingly, steatosis severity in patients with MASLD or MASH is evaluated based on CAP score threshold values (Table 2) [45,86,87].

**Figure 5:**
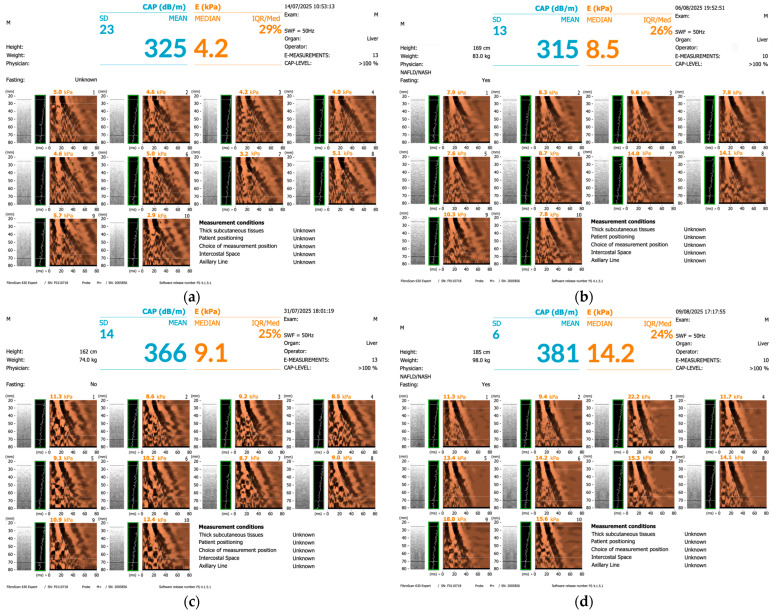
FibroScan images in patients with T2DM (original images courtesy of Anca Maria Amzolini and Ion Cristian Efrem): (**a**) S2 F0–F1; (**b**) S2 F2; (**c**) S3 F2; (**d**) S3 F4.

The fibrosis stage is assessed using LSM cutoff values (Table 3) [45,88]. LSM values typically range from 2.5 to 75 kPa [45,83]. Accurate interpretation depends on key reliability parameters, including the interquartile range (IQR), which indicates measurement variability and should remain below 30% [45,89], as well as the success rate, defined as the ratio of successful to total measurements, which should be not less than 60% [45,83,90]. Fibrosis severity in patients with MASLD or MASH is staged according to the NASH Clinical Research Network Scoring System, ranging from F0 to F4 (Figure 5) [86,90]. In addition, XL probes have been designed to facilitate evaluation of patients with higher levels of adiposity [45,88].

## 4. MASH Therapeutic Management

**Figure 6:**
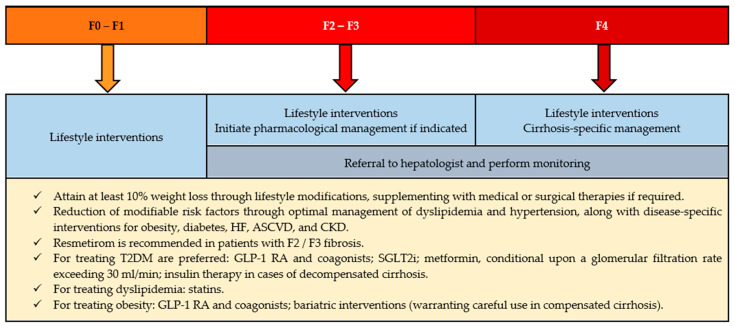
MASLD and MASH management. F0: fibrosis stage 0; F1: fibrosis stage 1; F2: fibrosis stage 2; F3: fibrosis stage 3; F4: fibrosis stage 4; HF: heart failure; ASCVD: atherosclerotic cardiovascular disease; CKD: chronic kidney disease; T2DM: type 2 diabetes mellitus; GLP-1 RA: glucagon-like peptide-1 receptor agonists; SGLT2i: sodium–glucose cotransporter-2 inhibitors (adapted from [67]).

As previously highlighted, accurately diagnosing and stratifying MASLD based on fibrosis risk is crucial to identify patients who may benefit from pharmacological treatment and those who can be managed only with lifestyle interventions (Figure 6) [67,75,91,92].

Management of patients with MASLD without evidence of MASH or significant fibrosis is centered primarily on lifestyle interventions, including smoking cessation, along with appropriate management of associated cardiovascular and renal risk factors. Patients with overweight or obesity should be encouraged to achieve at least 10% reduction in body weight through lifestyle interventions, with the addition of pharmacologic or surgical obesity treatments when indicated. Evaluation, conducted at least annually, should include systematic reassessment of progression to advanced hepatic disease [67].

### 4.1. Lifestyle Interventions: Diet and Physical Activity

Commitment to a healthy lifestyle, encompassing balanced nutrition and consistent physical activity, constitutes the first-line intervention in the management of both T2DM and MASLD. Energy-restricted diets and exercise interventions not only enhance glycemic control but also decrease hepatic steatosis and may even result in remission of MASH [25,93]. Consequently, lifestyle intervention represents the most evidence-based therapeutic approach for MASLD, although sustained long-term efficacy remains suboptimal [81,94]. MASH regression has been observed in approximately 58% of patients achieving weight loss greater than 5% of baseline body weight, with response rates increasing to nearly 90% among those attaining weight loss greater than 10% [81,95]. Nutritional strategies should be personalized, favoring hypocaloric regimens such as low-fat, low-carbohydrate, or Mediterranean dietary patterns. Additionally, isocaloric high-protein diets have demonstrated efficacy in reducing hepatic steatosis and inflammatory activity in patients with T2DM [81,96].

Physical activity has been associated with reductions in hepatic steatosis and improvements in liver stiffness [81,97], independent of dietary interventions [98]. Moderate-to-vigorous physical activity was shown to improve hepatic steatosis, with optimal benefits observed among individuals engaging in more than 250 min of exercise per week [81,99]. According to the 2024 European Association for the Study of the Liver (EASL)–European Association for the Study of Diabetes (EASD)–European Association for the Study of Obesity (EASO) guideline recommendations, patients should perform moderate-intensity physical activity for more than 150 min weekly, or vigorous-intensity exercise for over 75 min weekly, to mitigate hepatic steatosis. Furthermore, a body weight reduction exceeding 5% is associated with a reduction in hepatic steatosis, a 7–10% reduction is required to achieve improvements in hepatic inflammation, and a weight loss greater than 10% is necessary to ameliorate fibrosis [92]. Nonetheless, lifestyle interventions exert gradual effects, require sustained adherence over the medium to long term, may be undermined by factors such as psychological stress and sedentary behavior [81,95], and exercise alone does not significantly improve fibrosis or histological features [81,100].

### 4.2. Surgical Treatment

Bariatric surgery effectively promotes MASH resolution, with T2DM representing the only baseline factor adversely influencing MASH improvement in the absence of fibrosis progression [25,101]. Conversely, bariatric procedures may induce T2DM remission in early MASLD, emphasizing the role of hepatic injury in post-surgical metabolic outcomes [25,102]. Histological amelioration of ballooning and lobular inflammation occurs in about three-quarters of patients with steatohepatitis [81,103]. Among patients with obesity and MASH undergoing bariatric surgery, 84% experienced MASH resolution, 56% had fibrosis resolution, and 70% showed fibrosis regression five years after surgery [104,105]. Additionally, bariatric surgery markedly reduced the risk of major adverse hepatic and cardiovascular events in this population [104,106].

### 4.3. Thyroid Hormone Receptor β Agonists

Resmetirom is a liver-targeted, thyroid hormone receptor β-selective agonist, the first treatment that has received approval, representing a pivotal progress in managing adults with non-cirrhotic MASH and moderate to advanced fibrosis [104,107]. Alongside antisteatogenic effects, resmetirom exhibited increased circulating adiponectin levels and improvements in atherogenic lipid profile, including low-density lipoprotein cholesterol (LDL-c), TG, apolipoprotein B (ApoB), and ApoC-III [108,109,110]. No clinically meaningful effects were observed in body weight, glycemic control, heart rate, or blood pressure [107]. The ability of resmetirom to reduce hepatic lipid content, as well as plasma levels of atherogenic lipids and lipoproteins, was further supported by findings from the 52-week MAESTRO-NAFLD-1 phase 3 trial [111]. Notably, recent data from the phase 3 MAESTRO-NASH trial demonstrated that both primary histological endpoints, resolution of MASH and improvement in fibrosis, were achieved after 52 weeks of resmetirom therapy [107,108]. Following its FDA approval in the United States in March 2024, emerging real-world evidence has supported its tolerability and effectiveness in routine clinical practice, while also revealing limitations related to access and reimbursement. In Europe, a positive Committee for Medicinal Products for Human Use (CHMP) opinion was issued by the European Medicines Agency (EMA) in June 2025, with wider availability expected in the coming months [112].

Beyond resmetirom, two additional thyroid hormone receptor β selective agonists, ASC41 and VK2809, are currently undergoing evaluation in phase 2 randomized controlled trials (RCTs) [108].

### 4.4. Antidiabetic Treatment

Suboptimal glycemic and weight control hasten cirrhosis progression, highlighting the necessity for a holistic treatment strategy targeting multiple cardiometabolic contributors to MASLD that relies on pharmacological interventions with broad beneficial effects [113].

#### 4.4.1. Glucagon-like Peptide-1 Receptor Agonists (GLP-1 RA) and Coagonists

Growing evidence highlights the clinical relevance of GLP-1 RA as an effective therapy for T2DM and obesity [114,115]. Despite the absence of this receptor’s expression on hepatocytes, GLP-1 RA has been shown to exert indirect hepatic effects through improvements in IR, glycemic control, and body weight [114]. Beyond these metabolic benefits, GLP-1 signaling also plays a significant role in modulating inflammatory pathways, as well as exerting antifibrotic effects through inhibition of hepatic stellate cell activation, and reduction in oxidative stress and profibrotic cytokine release [116].

Semaglutide, a GLP-1 RA, was approved by the FDA in September 2025 for the treatment of adults with MASH and moderate to advanced fibrosis [117]. Interim histology-based results, at week 72, demonstrated that 62.9% of participants treated with once-weekly subcutaneous semaglutide administered at a dose of 2.4 mg achieved MASH resolution without worsening fibrosis, in comparison with 34.3% in the placebo group, while fibrosis improvement without steatohepatitis worsening occurred in 36.8% versus 22.4% of participants, respectively. Furthermore, concurrent steatohepatitis resolution and fibrosis improvement were achieved in 32.7% of patients receiving semaglutide, compared to 16.1% of placebo-treated patients. Participants treated with semaglutide experienced a mean body weight decrease of 10.5%, and 2.0% in those receiving placebo [118]. The ESSENCE phase 3 clinical trial is ongoing and will continue for a total duration of 240 weeks to assess whether the histological improvements in inflammation and fibrosis observed at 72 weeks translate into clinically meaningful reductions in mortality, liver transplantation, and other liver-related outcomes [117,118]. Recent cohort data represent the first direct comparison of GLP-1 RA and long-acting insulin treatment in T2DM, indicating a decreased risk of cirrhosis and HCC among patients receiving GLP-1 RA [119,120].

Another GLP-1 RA, liraglutide, administered at 1.8 mg daily, was demonstrated to induce MASH resolution compared with placebo, while fibrosis progression occurred in 9% of patients receiving liraglutide versus 36% of those in the placebo group in the LEAN phase 2 trial [81,121].

Emerging therapeutic strategies include dual and triple incretin agonists combining GLP-1 with glucagon or glucose-dependent insulinotropic polypeptide (GIP) signaling to potentiate metabolic outcomes [122].

Tirzepatide is the first single-molecule agonist with combined GLP-1 and GIP receptor activity authorized for the treatment of T2DM and obesity [104]. In a post hoc analysis of a phase 2 trial, the effects of once-weekly tirzepatide at doses of 1, 5, 10, and 15 mg on MASH-related biomarkers were evaluated over 26 weeks in patients with T2DM versus once-weekly dulaglutide 1.5 mg and placebo. Higher tirzepatide doses were linked to improvements in several biomarkers [104,123]. Data from a SURPASS-3 phase 3 sub-study demonstrated that 52 weeks of tirzepatide treatment was associated with significantly greater reductions in hepatic fat content compared with insulin degludec [124]. Tirzepatide is being investigated in the ongoing phase 2b SYNERGY-NASH trial in patients with MASH and stage 2 and 3 fibrosis, with or without T2DM [104].

In a 54-week trial, once-daily cotadutide, a GLP-1/glucagon dual receptor agonist, demonstrated improvements in MASH-related biomarkers, as well as in FIB-4 and NAFLD Fibrosis Score, compared with placebo [104,125]. Evidence from a 12-week randomized, double-blind, placebo-controlled study indicates that pemvidutide, another GLP-1/glucagon receptor agonist, effectively reduces hepatic steatosis, inflammatory biomarkers, and body weight in patients with MASH [114,126]. Similarly, survodutide induced substantial weight loss and achieved histological MASH resolution alongside fibrosis improvement after one year of therapy [114,127]. Efinopegdutide demonstrated significantly greater relative hepatic fat reductions compared with semaglutide 1.0 mg in an open-label phase 2a study [104,128]. Efinopegdutide is being investigated in a phase 2 trial comparing its effects with placebo and semaglutide 2.4 mg on liver histology in individuals with MASH and stage 2 or stage 3 fibrosis [104]. Evidence suggests that mazdutide may be effective in addressing obesity, diabetes, and hepatic complications, including fibrosis and steatosis [113,129].

Retatrutide, a triple GLP-1, GIP, and glucagon receptor coagonist, is under investigation for obesity management. In a 48-week phase 2 study, once-weekly retatrutide at doses of 1, 4, 8, and 12 mg produced significant weight loss up to 24.2% compared with placebo [104,130]. Relative hepatic fat reductions at 24 weeks were nonlinearly related to weight loss, approaching 82.4% among participants treated with retatrutide 12 mg [104,131].

Efocipegtrutide, a GLP-1/GIP/glucagon triple receptor agonist, is currently under investigation for MASH. Phase 1b/2a data in patients with obesity and MASLD without T2DM [104,132] showed that once-weekly eficopegtrutide produced significantly greater relative reductions in hepatic fat compared with placebo after 8 weeks. A phase 2 trial assessing its impact on liver histology in patients with MASH and fibrosis stages 1–3 is ongoing [133].

#### 4.4.2. Long-Acting Amylin Analogs

Cagrilintide has been assessed for its therapeutic potential in obesity and T2DM and is currently under investigation for its effects compared with semaglutide or placebo in individuals with MASH and substantial fibrosis or cirrhosis [113].

#### 4.4.3. Sodium–Glucose Cotransporter-2 Inhibitors (SGLT2i)

SGLT2i acts by preventing glucose reabsorption in the proximal renal tubules, resulting in glucosuria, improved insulin sensitivity, and weight loss. These metabolic effects may translate into reduced inflammation and hepatic steatosis, potentially mediated by increased adipose lipolysis, lower ectopic fat deposition, and regulation of hepatic adenosine monophosphate (AMP)-activated protein kinase activity [134,135].

Evidence from preclinical and early-phase clinical studies, including dapagliflozin and empagliflozin trials, suggests beneficial effects on liver enzymes, hepatic steatosis, and fibrosis-associated biomarkers [136,137]. It is worth noting that dapagliflozin represents the most advanced therapy under investigation, specifically in patients with MASH, in a phase 3 trial with complete enrollment, and results pending publication [9,134]. A small pilot study showed that empagliflozin was associated with reductions in steatosis, hepatocyte ballooning, and fibrosis after 24 weeks of therapy in individuals with T2DM and MASH, compared with baseline and a historical control group [108,138]. Similarly, canagliflozin treatment resulted in improvement across all MASH histological components, including fibrosis, after one year compared with baseline, with sustained benefits over five years [108,139]. Consistently, a 72-week randomized trial of ipragliflozin in individuals with T2DM and MASLD demonstrated greater MASH resolution and fibrosis regression, with NAFLD activity score improvement mainly attributable to reduced hepatocyte ballooning [108,140].

#### 4.4.4. Metformin

Although early small, uncontrolled studies suggested that metformin reduces liver enzyme levels and improves insulin sensitivity, robust evidence for histological improvement in MASH is lacking. Nevertheless, observational studies support continued metformin use in patients with T2DM and MASLD-driven advanced fibrosis or cirrhosis due to associations with improved survival and reduced cancer risk, unless contraindicated [92].

#### 4.4.5. Insulin

While no placebo-controlled trials specifically assessed insulin therapy in MASH, several studies incorporated insulin-based treatment arms. These studies neither assessed histological liver improvement nor demonstrated significant reductions in hepatic steatosis, body weight, or liver enzymes [58].

### 4.5. Peroxisome Proliferator-Activated Receptor (PPAR) Agonists

PPAR plays a pivotal role in the regulation of fatty acid metabolism, inflammatory processes, and fibrogenesis [108].

Pioglitazone, a PPARγ agonist, improves insulin sensitivity and has been shown to reduce insulin resistance, hepatic steatosis, alongside histological improvements in inflammation and hepatocyte ballooning [119]. Pooled network meta-analysis data indicated that pioglitazone outperformed placebo in promoting MASH resolution and improving fibrosis [12,141]. However, its clinical use is constrained by notable adverse effects, including weight gain, increased risk of osteoporosis among postmenopausal women, a debated association with bladder cancer, and exacerbation of heart failure in patients with underlying cardiac disease [12,119]. Recent phase 2 randomized controlled trial (RCT) data indicate that PXL065, a deuterium-stabilized R-pioglitazone enantiomer, provides a comparable reduction in hepatic lipid content, along with MASH and fibrosis improvements with a reduced burden of PPARγ-typical adverse effects in individuals with MASH [108,142,143].

Saroglitazar is a dual PPARα/γ agonist and was associated with significant improvement in IR, steatosis, and liver enzymes [81,144]. Clinical trials assessing liver histology are currently in progress [92].

Lanifibranor, a pan-PPAR agonist, has demonstrated dose-dependent MASH resolution alongside regression of hepatic fibrosis stage and is currently under investigation in a phase 3 trial [81,145].

### 4.6. Fibroblast Growth Factor (FGF) Analogs

FGF21 is a metabolic hormone with a central role in the regulation of energy balance, glucose homeostasis, and lipid metabolism [142].

The long-acting FGF21 analog, pegozafermin, is being investigated as a potential therapy for MASH and severe hypertriglyceridemia. In a phase 2b trial, fibrosis improvement of at least one stage without worsening steatohepatitis and MASH resolution without fibrosis progression was achieved [31,146]. The phase 3 study will assess long-term effects on fibrosis regression in patients with MASH and compensated cirrhosis (F4 stage) over 24 months [31,113].

Efruxifermin represents another long-acting FGF21 analog under investigation. Results from a phase 2b trial confirmed at least one-stage fibrosis improvement without worsening steatohepatitis [31,147]. Ongoing phase 3 trials aim to further evaluate the efficacy of efruxifermin in MASLD patients with varying degrees of fibrosis [31].

Aldafermin is a FGF19 analog with inhibitory effects on bile acid production and regulatory effects on metabolic homeostasis [113]. Available studies have revealed beneficial effects on hepatic steatosis, but have not yet demonstrated a significant improvement in histologic fibrosis [31,148,149].

### 4.7. Farnesoid X Receptor (FXR) Agonist

As a bile acid-sensing nuclear receptor predominantly expressed in the liver and intestine, FXR plays a central role in MASH pathogenesis by regulating gene networks involved in cholesterol and bile acid homeostasis, hepatic gluconeogenesis and lipogenesis, and also exerts important effects on inflammatory pathways [81,114,150].

Obeticholic acid (OCA) is the most extensively studied FXR agonist. Despite demonstrating antifibrotic efficacy in a phase 3 trial [151], failure to achieve MASH resolution and unfavorable safety concerns resulted in the premature discontinuation of its development for MASH [114].

In a phase 2 clinical trial, tropifexor was evaluated in patients with MASH and stage 1–3 fibrosis. Early results demonstrated safety and significant reductions in hepatic steatosis, liver enzyme levels, and body weight [81,152].

However, cilofexor reduced hepatic steatosis and decreased primary bile acid levels without significantly affecting fibrosis in a phase 2 trial [81,153].

Vonafexor demonstrated significant reductions in hepatic fat content, liver enzyme levels, and body weight in a phase 2 study, highlighting its potential as a valuable therapeutic candidate in ongoing MASH research [33,154].

Several other FXR agonists are undergoing clinical assessment. EDP305 demonstrated reductions in hepatic steatosis in non-cirrhotic MASH in a phase 2 study and is currently being evaluated in patients with MASH and stage 2 and stage 3 fibrosis in a phase 2b trial [81,155]. In a phase 2 study, TERN-101 reduced liver enzymes in patients with stage 1 to 3 fibrosis and improved inflammation and fibrosis [81].

### 4.8. Lipogenesis Inhibitors

#### 4.8.1. Stearoyl-Coenzyme A Desaturase 1 (SCD1) Modulators

SCD1 modulators decrease monounsaturated fatty acid production, thereby attenuating lipotoxicity [33]. In a phase 2b trial, Aramchol was associated with resolution of steatohepatitis without fibrosis progression and reduction in fibrosis severity by at least one stage without worsening MASH [9,31,156]. Encouraging outcomes led to the initiation of the phase 3 trial, which was subsequently suspended following an interim open-label analysis that confirmed improvement across histology, imaging, and biomarker endpoints [9].

#### 4.8.2. Acetyl-Coenzyme A Carboxylase (ACC) Inhibitors

In a RCT performed in individuals with MASLD, firsocostat, an ACC inhibitor, produced a moderate reduction in hepatic steatosis and improved liver fibrosis, although it also caused an elevation in plasma TG [81,108]. Another ACC inhibitor, clesacostat, demonstrated greater reduction in hepatic steatosis, although it also led to higher plasma TG elevations [108]. Nevertheless, safety concerns prompted the premature discontinuation of clesacostat monotherapy development [9].

PF-05221304 is a highly potent, reversible inhibitor targeting ACC1/2 that reduced hepatic lipid accumulation as much as 65% in a phase 2 trial [33,81,157].

#### 4.8.3. Diacylglycerol Acyltransferase 2 (DGAT2) Inhibitors

Ervogastat effectively reduces hepatic fat and, unlike ACC inhibitors, additionally lowers plasma TG levels [9,158].

Phase 2 studies demonstrated that ION224 improved liver histology without fibrosis progression, highlighting its potential to reduce hepatic steatosis and inflammation in MASH [33].

#### 4.8.4. Fatty Acid Synthase (FASN) Inhibitors

FASN inhibitors suppress DNL, thereby mitigating lipotoxicity, hepatic inflammation, and fibrosis. After leading to a moderately reduced hepatic steatosis, with concomitant improvements in biochemical, inflammatory, and fibrosis-related biomarkers in patients with MASH in a phase 2a study [159], denifanstat achieved notable MASH resolution in phase 2b clinical trial and obtained FDA breakthrough designation [134,160].

### 4.9. Antifibrotic and Anti-Inflammatory Agents

In a phase 2b study, cenicriviroc, a dual chemokine receptor 2 (CCR2) and chemokine receptor 5 (CCR5) antagonist, improved liver fibrosis without worsening MASH, although no significant histological MASH resolution was observed [81,113,161]. However, the subsequent phase 3 clinical trial was stopped ahead of schedule after an interim analysis failed to demonstrate efficacy [113,162].

Belapectin, a galectin-3 inhibitor, reduced hepatic venous pressure gradient and showed potential for preventing esophageal varices in patients with compensated MASH cirrhosis in a phase 2a trial subgroup analysis [33,163]. Subsequently, a phase 2b/3 study was initiated to further investigate its clinical benefits [33,113].

Selonsertib, an apoptosis signal-regulating kinase 1 (ASK1) inhibitor, demonstrated significant reductions in hepatic fibrosis, apoptosis, and necrosis serum biomarkers, and improvements in liver enzyme profiles [33]. Although it was well tolerated in two phase 3 clinical trials, selonsertib failed to improve hepatic fibrosis without exacerbating MASH among participants with stage 3 fibrosis or compensated cirrhosis, and both studies were halted ahead of schedule due to lack of efficacy [9,33,81,164].

Although preliminary studies indicated ursodeoxycholic acid (UDCA) efficacy, subsequent RCT was unable to demonstrate significant histological benefit in patients with MASH [12,165,166]. Norursodeoxycholic acid, a synthetic UDCA analog, exhibits antifibrotic, anti-inflammatory, and anticholestatic effects in preclinical studies. A recent phase 2 trial suggests improvements in hepatic steatosis and liver enzymes [92,167]. A subsequent phase 2b trial enrolling patients with biopsy-confirmed MASH is underway to assess its efficacy in achieving histological improvement [168]. Berberine ursodeoxycholate, an ionic salt composed of berberine and ursodeoxycholic acid, was associated with a reduction in hepatic steatosis, alongside improvements in glucose homeostasis and hepatic enzyme levels, in patients with MASH and T2DM, in a phase 2 study [52,169].

Silymarin exhibited a favorable safety and tolerability profile, may reduce liver enzyme levels, but no significant histological improvement was observed in phase 2 clinical trials [12,92].

Rencofilstat, an analog of cyclosporine A, exerts antifibrotic effects. Its therapeutic potential was demonstrated in a phase 2b study in individuals with MASH and advanced fibrosis (stages F2,3), rencofilstat promoting hepatic function improvement and attenuating portal-systemic shunting [168,170].

### 4.10. Lipid-Lowering Agents

Evidence from recent clinical trials indicates that statin therapy may decrease hepatic fat accumulation and fibrosis, and ameliorate MASLD and MASH manifestations [81,171]. Yet, the absence of large RCTs with histological endpoints prevents establishing statins as a treatment for MASH; similar limitations apply to ezetimibe and fibrates [12,92].

RCTs have shown that ethyl eicosapentaenoic acid supplementation failed to achieve histological improvement compared with placebo [12,92,172]. Ongoing studies are evaluating a structurally engineered omega-3 fatty acid. A phase 2b trial evaluated icosabutate, an oral semisynthetic free fatty acid receptor 1 (FFAR1) free fatty acid receptor 4 (FFAR4) agonist, showing a potential beneficial effect on surrogate histological endpoints, notably fibrosis regression, despite not achieving the prespecified primary endpoint of MASH resolution without progression of fibrosis [114,173].

### 4.11. Vitamin E

Vitamin E is a lipid-soluble vitamin with antioxidant properties that exhibits anti-inflammatory and anti-apoptotic effects. In addition, it inhibits DNL, thereby contributing to a reduction in hepatic lipid accumulation [92]. Observational case–control studies indicate that prolonged vitamin E exposure may reduce the risk of mortality, liver transplantation, and hepatic decompensation in patients with MASH and advanced fibrosis or cirrhosis [92,174]. Although the largest RCT demonstrated improvements in steatohepatitis, steatosis, lobular inflammation, and hepatic enzymes with vitamin E supplementation at 800 IU/day for a duration of 2 years in MASH among patients without T2DM, data supporting fibrosis improvement are inconclusive [31,92,175]. Given the limited availability of effective therapies prior to recent developments, this agent has been used for MASH in individuals without T2DM. Nonetheless, due to the absence of large-scale phase 3 trials, vitamin E is no longer recommended in the latest clinical guidelines [31,92].

### 4.12. Probiotics

The bidirectional relationship between MASH and gut microbiota highlights the potential of microbiome-targeted interventions, including probiotics, prebiotics, and synbiotics, or fecal microbiota transplantation, which may provide a novel therapeutic approach, targeting dysbiosis-driven liver inflammation and damage [176]. Probiotics, specifically *Lactobacillus* and *Bifidobacterium* species, have been associated with steatosis reduction and liver enzyme improvement. Synbiotics, combining probiotics with prebiotics such as fructo-oligosaccharides and inulin, provide an enhanced strategy by supporting the growth of beneficial intestinal bacteria, thereby optimizing microbiota composition and decreasing hepatic steatosis [33,177]. Another emerging approach, fecal microbiota transplantation, involves fecal material transfer from healthy donors to re-establish intestinal microbiome balance, with initial clinical trials reporting enhanced insulin sensitivity and reduced hepatic content [33,178].

### 4.13. Gene Targeting

GSK4532990, a small interfering RNA therapeutic directed against HSD17B13, is presently being investigated for safety and efficacy in a phase 2b clinical trial involving NASH patients with advanced fibrosis (stage 3) [168].

AZD2693 was initially under investigation in phase 2 trials to determine its safety profile and therapeutic efficacy in decreasing hepatic fat content and fibrosis in individuals with a genetic predisposition; however, development was discontinued following disappointing outcomes in the Phase 2b clinical trial, as reported in late 2025 [33,179].

Given the breadth of therapeutic agents discussed, Table 4 summarizes the most clinically relevant and advanced therapies supported by evidence from randomized controlled trials and phase 2–3 clinical studies, while additional compounds are described in the main text.

Due to the multifactorial nature of MASH, using drug combinations could provide greater therapeutic benefit through synergistic or complementary effects and enhance safety by minimizing the dose of each drug [168]. Semaglutide in combination with cilofexor (FXR agonist) and/or firsocostat (ACC inhibitor) in patients with compensated MASH cirrhosis provided superior benefits for hepatic steatosis and fibrosis biomarkers relative to semaglutide alone [52,180]. Treatment with combined cilofexor and firsocostat in patients with cirrhosis or bridging fibrosis led to superior NASH activity improvements versus placebo or individual agents [52,181]. The combination of ACC and DGAT2 inhibitors effectively lowered hepatic fat and mitigated the side effect of elevated TG [52,157].

Furthermore, cardiovascular disease represents the leading cause of morbidity and mortality in patients with MASH, a burden that is largely attributable to shared cardiometabolic risk factors, including insulin resistance, type 2 diabetes mellitus, dyslipidemia, and obesity [14,31]. Beyond their hepatic effects, several therapeutic agents discussed in this section—such as GLP-1 receptor agonists, sodium–glucose cotransporter-2 inhibitors, and thyroid hormone receptor β agonists—have demonstrated favorable effects on cardiovascular risk factors, including body weight, glycemic control, and atherogenic lipid profiles [182,183]. Accordingly, cardiovascular considerations are essential when evaluating emerging MASH therapies, particularly in patients with coexisting metabolic disease, as an integrated cardio-hepatic approach may improve overall clinical outcomes.

## 5. Conclusions

Prompt diagnosis and early clinical management play a pivotal role in improving patient prognosis, given the natural history and progressive course of MASH. Research into non-invasive or serum biomarker approaches holds promise for enhancing diagnostic precision, while the identification of predictive markers may optimize personalized therapeutic interventions.

Clinical insights derived from RCTs are expected to improve the quality of life and long-term outcomes in patients with MASH. Pending comprehensive evidence, prompt action is necessary to mitigate fibrosis advancement and cirrhosis development. This strategy, best implemented by multidisciplinary medical teams, involves T2DM treatments capable of improving steatohepatitis and preventing fibrosis progression, alongside obesity management through lifestyle modification and pharmacotherapy.

## Figures and Tables

**Table 1:** MASLD definition.

Hepatic Steatosis (≥5%) + One of the Three Criteria	
1. Overweight Obesity	
2. T2DM	
3. Evidence of metabolic dysregulation	Abdominal obesity
	Hyperglycemia
	Hypertension
	Elevated TG
	Low HDL-c

T2DM: type 2 diabetes mellitus; TG: triglycerides; HDL-c: high-density lipoprotein cholesterol.

**Table 2:** Steatosis grade and CAP score ranges.

Steatosis Grade (Proportion of Affected Hepatocytes)	CAP Score (dB/m)
S0 (<5%)	≤293
S1 (5–33%)	294–309
S2 (34–66%)	310–330
S3 (>66%)	≥331

S0: absent steatosis; S1: mild hepatic fat accumulation; S2: moderate fat accumulation; S3: extensive hepatic steatosis (adapted from [45]).

**Table 3:** Fibrosis severity and LSM value ranges.

Fibrosis Severity	LSM Value (kPa)
F0–F1	≤8.1
F2	8.2–9.6
F3	9.7–13.5
F4	≥13.6

F0: absent fibrosis; F1: mild fibrosis involving perisinusoidal or portal areas; F2: moderate fibrosis with both perisinusoidal and portal or periportal involvement; F3: advanced fibrosis characterized by septal or bridging fibrosis; F4: cirrhosis (adapted from [45]).

**Table 4:** Summary of selected pharmacological therapies for MASH, clinical trial phase, and reported outcomes.

Therapeutic Category	Agent(s) Mentioned	Clinical Trial Phase Stated	Outcomes Stated in Text	Trial Status Stated
Thyroid hormone receptor β agonists	Resmetirom	Phase 3	Reduction in hepatic lipid content; improvement in atherogenic lipid profile; resolution of MASH and improvement in fibrosis after 52 weeks	FDA approved (March 2024)
	ASC41; VK2809	Phase 2 randomized controlled trials	Under evaluation	Ongoing
GLP-1 receptor agonists	Semaglutide	Phase 3	MASH resolution without worsening fibrosis; fibrosis improvement without steatohepatitis worsening; concurrent steatohepatitis resolution and fibrosis improvement; body weight reduction	FDA approved (September 2025); Phase 3 ongoing
	Liraglutide	Phase 2	Induced MASH resolution; lower fibrosis progression compared with placebo	Completed
Dual GLP-1/glucagon receptor agonists	Tirzepatide	Phase 2; Phase 2b	Improvement in MASH-related biomarkers; reduction in hepatic fat content	Phase 2b ongoing
	Cotadutide	Phase 2	Improvement in MASH-related biomarkers; improvement in FIB-4 and NAFLD Fibrosis Score	Completed
	Pemvidutide	Phase 1b/2a	Reduction in hepatic steatosis, inflammatory biomarkers, and body weight	Completed
	Survodutide	Phase 2	Histological MASH resolution and fibrosis improvement after one year	Ongoing
	Efinopegdutide	Phase 2a; Phase 2	Greater relative hepatic fat reductions compared with semaglutide; effects on liver histology under investigation	Phase 2 ongoing
Triple GLP-1/GIP/glucagon receptor agonists	Retatrutide	Phase 2	Significant weight loss; relative hepatic fat reduction approaching 82.4%	Ongoing
	Efocipegtrutide	Phase 1b/2a; Phase 2	Greater relative reductions in hepatic fat compared with placebo	Phase 2 ongoing
SGLT2 inhibitors	Dapagliflozin	Phase 3	Beneficial effects on liver enzymes, hepatic steatosis, and fibrosis-associated biomarkers	Enrollment completed; results pending
	Empagliflozin	Pilot study	Reduction in steatosis, hepatocyte ballooning, and fibrosis after 24 weeks	Completed
	Canagliflozin	Observational study	Improvement across all MASH histological components, including fibrosis	Completed
	Ipragliflozin	Randomized trial (72 weeks)	Greater MASH resolution and fibrosis regression; NAFLD activity score improvement mainly due to reduced hepatocyte ballooning	Completed
PPARγ agonist	PXL065	Phase 2	Reduction in hepatic lipid content; MASH and fibrosis improvements with fewer PPARγ-typical adverse effects	Ongoing
Pan-PPAR agonist	Lanifibranor	Phase 3	Dose-dependent MASH resolution and regression of hepatic fibrosis stage	Phase 3 ongoing
Gene targeting	GSK4532990	Phase 2b	Safety and efficacy under investigation in patients with advanced fibrosis (stage 3)	Ongoing
	AZD2693	Phase 2; Phase 2b	Intended to reduce hepatic fat content and fibrosis; disappointing outcomes in phase 2b	Discontinued (late 2025)

FDA: Food and Drug Administration; FIB-4: Fibrosis-4 index; MASH: metabolic dysfunction-associated steatohepatitis; NAFLD: nonalcoholic fatty liver disease; PPARγ: peroxisome proliferator-activator receptor γ; SGLT2: sodium–glucose cotransporter-2.

## Data Availability

No new data were created or analyzed in this study. Data sharing is not applicable to this article.

## Abbreviations

The following abbreviations are used in this manuscript:

A1AT	alpha-1 antitrypsin
ACC	acetyl-coenzyme A carboxylase
ADN	deoxyribonucleic acid
AIP	atherogenic index of plasma
ALT	alanine aminotransferase
AMA	anti-mitochondrial antibody
AMP	adenosine monophosphate
ANA	antinuclear antibody
ApoB	apolipoprotein B
ApoC-III	apolipoprotein C-III
ASCVD	atherosclerotic cardiovascular disease
ASK1	apoptosis signal-regulating kinase 1
ASMA	anti-smooth muscle antibody
AST	aspartate aminotransferase
CAP	controlled attenuation parameter
CCR2	chemokine receptor 2
CCR5	chemokine receptor 5
CHMP	Committee for Medicinal Products for Human Use
CKD	chronic kidney disease
CRP	C-reactive protein
dB/m	decibels per meter
DGAT2	diacylglycerol acyltransferase 2
DNL	de novo lipogenesis
EASD	European Association for the Study of Diabetes
EASL	European Association for the Study of the Liver
EASO	European Association for the Study of Obesity
EMA	European Medicines Agency
FASN	fatty acid synthase
FATP5	fatty acid transport protein-5
FDA	Food and Drug Administration
FFAR1	free fatty acid receptor 1
FFAR4	free fatty acid receptor 4
FFAs	free fatty acids
FGF	fibroblast growth factor
FIB-4	Fibrosis-4 index
FXR	farnesoid X receptor
GLP-1 RA	glucagon-like peptide-1 receptor agonists
HBcAb	hepatitis B core antibody
HBsAb	hepatitis B surface antibody
HBsAg	hepatitis B surface antigen
HCC	hepatocellular carcinoma
HCV	hepatitis C virus
HDL-c	high-density lipoprotein cholesterol
HF	heart failure
HOMA-IR	homeostasis model assessment of insulin resistance
HSD17B13	17-beta hydroxysteroid dehydrogenase 13 gene
IL-1β	interleukin-1 beta
IL-6	interleukin 6
IR	insulin resistance
kPa	kilopascals
LDL-c	low-density lipoprotein cholesterol
LPL	lipoprotein lipase
LPS	lipopolysaccharides
LSM	liver stiffness measurement
MAFLD	metabolic-associated fatty liver disease
MASH	metabolic dysfunction-associated steatohepatitis
MASLD	metabolic dysfunction-associated steatotic liver disease
MBOAT7	membrane-bound O-acyltransferase domain-containing protein 7
MetS	metabolic syndrome
MHz	megahertz
NAFLD	nonalcoholic fatty liver disease
NASH	nonalcoholic steatohepatitis
NHHR	non-HDL-c-to-HDL-c ratio
OCA	obeticholic acid
PNPLA3	patatin-like phospholipase domain containing 3
PPAR	peroxisome proliferator-activator receptor
RCT	randomized controlled trial
RNA	ribonucleic acid
ROS	reactive oxygen species
SCD1	stearoyl-coenzyme A desaturase 1
SGLT2i	Sodium–glucose cotransporter-2 inhibitors
T2DM	type 2 diabetes mellitus
TG	triglycerides
TLR	toll-like receptor
TM6SF2	transmembrane 6 superfamily member 2
TMAO	trimethylamine N-oxide
TNF-α	tumor necrosis factor alpha
TyG	triglyceride-glucose
UDCA	ursodeoxycholic acid
